# Effect of Low-Level Laser Therapy (LLLT) in Pulmonary Inflammation in Asthma Induced by House Dust Mite (HDM): Dosimetry Study

**DOI:** 10.1155/2019/3945496

**Published:** 2019-03-21

**Authors:** Nicole Cristine Rigonato-Oliveira, Auriléia Aparecida de Brito, Luana Beatriz Vitoretti, Gabriel de Cunha Moraes, Tawany Gonçalves, Karine Zanella Herculano, Cintia Estefano Alves, Adriana Lino-dos-Santos-Franco, Flávio Aimbire, Rodolfo Paula Vieira, Ana Paula Ligeiro de Oliveira

**Affiliations:** ^1^Post-Graduate Program in Biophotonics Applied to Health Sciences, University Nove de Julho (UNINOVE), Sao Paulo, Brazil; ^2^Heart Institute of Clinics Hospital, Medical School of the University of São Paulo (FMUSP), São Paulo, Brazil; ^3^Post-Graduate in Public Heath, University Nove de Julho (UNINOVE), São Paulo, Brazil; ^4^Biotechnology and Translational Medicine, Federal University of São Paulo–UNIFESP, Sao José dos Campos, Brazil; ^5^Universidade Brasil, Post-Graduation Program in Bioengineering and in Biomedical Engineering, Campus Itaquera, Rua Carolina Fonseca 235, São Paulo, SP 08230-030, Brazil; ^6^Brazilian Institute of Teaching and Research in Pulmonary and Exercise Immunology (IBEPIPE), Rua Pedro Ernesto 240, São José dos Campos, SP 12245-520, Brazil; ^7^Federal University of São Paulo (UNIFESP), Post-Graduation Program in Sciences of Human Movement and Rehabilitation, Avenida Ana Costa 95, Santos, SP 11060-001, Brazil; ^8^Anhembi Morumbi University, School of Medicine, Avenida Deputado Benedito Matarazo 4050, São José dos Campos, SP 12230-002, Brazil

## Abstract

Asthma is characterized by chronic inflammation in the airways. Several models have been proposed for the discovery of new therapies. Low-Level Laser Therapy (LLLT) is relatively new and effective, very low cost, with no side effects. However, there is still no consensus on the optimal dose to be used. In this sense, the objective of the present study was to evaluate the best dose in an experimental model of asthma induced by House Dust Mite (HDM). Balb/c mice received administration of 100 ug/animal HDM and LLLT applications (diode laser: 660 nm, 100 mW and four different energies 1J, 3J, 5J, and 7.5J) for 16 days. After 24 hours, we studied inflammatory, functional, and structural parameters. The results showed that LBI was able to modulate the pulmonary inflammation observed by reducing the number of cells in Bronchoalveolar Lavage Fluid (BALF) as well as reducing the percentage of neutrophils, eosinophils and T lymphocytes. On the other hand, LLLT increased the level of IL-10 and reduced levels of IL-4, IL-5 and IL-13 in BALF. LLLT was able to reduce the production of mucus, peribronchial eosinophils, collagen deposition, bronchoconstriction index, and bronchial and muscular thickening in the airways. We concluded that the use of LLLT in the treatment of chronic inflammation of the airways attenuated the inflammatory process and functional and structural parameters. We emphasize, in general, that the 1J and 3J laser presented better results. Thus, photobiomodulation may be considered a promising tool for the treatment of chronic pulmonary allergic inflammation observed in asthma.

## 1. Introduction

Asthma is the most common chronic diseases worldwide. It is defined as a heterogeneous disease, characterized by chronic inflammation of the airways, which causes symptoms such as wheezing, shortness of breath, tightness in the chest, and cough, which vary over time in their occurrence, frequency, and intensity. According to a report published by the Global Initiative for Asthma in 2018, stated that approximately 300 million people in the world currently have asthma [1]. The mortality rate is estimated at 346,000 deaths per year [2].

The use of low-intensity laser has been studied since the 1960s [3]. Although low-intensity laser therapy is widely used in different lung diseases, there is still no consensus as to the best dose to be used in chronic asthma. Several studies are being carried out to evaluate the optimum dose of application, as well as the real effects of this irradiation on the tissues.

A growing number of studies in experimental models have demonstrated that LLLT may be a low cost and effective option to aid in the treatment of inflammatory and fibrotic diseases in general. These studies demonstrate the efficacy of laser in acute inflammatory pulmonary diseases [4, 5], chronic fibrotic pulmonary diseases [6, 7], and experimental asthma using ovalbumin [8–10]. Some studies have used LLLT in the clinical area and as a result have observed improvement in spirometric parameters, reduction of exhaled nitric oxide concentration, improved control of asthma exacerbation, and consequent reduction in drug use [11, 12].

Inflammation of the airways in asthma is characterized by the activation of Thelper 2 (Th2) cells and eosinophilia [13]. During the inflammatory process, proinflammatory cell and cytokine levels may increase markedly and IL-10 levels, on the other hand, are reduced. In this way, the use of photobiomodulation, especially Treg cells, is gaining important prominence in the LBI pathway, since they actively participate in the production of IL-10 [14].

In the present study, the data show that LLLT is able to act in the reduction of the local inflammatory process, reduction of the peribronchial infiltrate, and deposition of mucus and collagen in the airways of mice with asthma induced by HDM. In contrast, the findings show an increase in IL-10 in the groups submitted to laser treatment, thus suggesting that the inflammatory response may have been suppressed by the action of the anti-inflammatory cytokine IL-10.

## 2. Materials and Methods

### 2.1. Animals

Male Balb/c mice (weight range 22-25 g, 7 weeks old) were obtained from the University Nove de Julho and maintained in a 12 h light/dark cycle, with a controlled temperature (22  ±  3°C) and humidity at 50 ±  5%, with free access to rodent food and water. All experiments carried out in this study were approved by the Animal Care Committee Nove de Julho University.

### 2.2. Experimental Groups

The animals were divided into ten experimental groups: Basal (Unmanned animals), HDM (Animals sensitized and challenged with HDM), LLLT (Animals undergoing laser therapy), and HDM+LLLT (Animals sensitized and challenged with HDM+ animals undergoing laser therapy).

### 2.3. Allergic Pulmonary Inflammation with HDM

The animals were anesthetized with ketamine (100 mg/kg) and xylazine (10 mg/kg) and then 100 ug of the Der p extract (GreerLaboratories, Lenoir, NC) were dissolved in 30 *μ*l PBS and administered orotracheally on days 0 and 14, followed by 3 administrations per week until day 56.

### 2.4. Low-Level Laser Treatment (LLLT) Protocol

Diode laser (power 30 mW, 660 nm of wavelength, area 0,045 J/cm^2^ and energy density of 1J, 3 J, 5J and 7,5J) was used. LLLT was performed three times a week, for five weeks, starting from day 21 up to day 56. One hour after each HDM administration, LLLT was performed on the trachea, right wolf, and left wolf, using a small spot size (0.785 cm^2^) for 10, 30, 50, and 75 seconds per point, respectively.

### 2.5. Bronchoalveolar Lavage Fluid (BALF)

After anesthesia, the animals were cannulated, and the lungs were washed with 1.5 ml PBS (3x of 0.5 ml). The volume recovered was centrifuged at 1000 rpm at 4°C for 5 minutes. The supernatant used for analysis of the cytokines by the ELISA method. The cell button was resuspended in 1 ml of PBS and determination of the total cell number in the FLBA was performed by counting in the Neubauer Chamber. Aliquots of the resuspended material were used to prepare cytospin slides which were stained with May-Grunwald-Giemsa for differential count cells [15].

### 2.6. Evaluation of Cytokine Levels in BALF by ELISA

The levels of cytokines (IL-4, IL-5, IL-10, and IL-13) in the BALF supernatant and lung tissue homogenate were evaluated using the RD Standard Sets and Biolegend kits as instructed by the manufacturer. For the reading of the plate, SpectraMax i3 (Molecular Devices) equipment with adjusted absorbance of 450 nm was used.

### 2.7. Evaluation of Airway Inflammation and Remodeling by Histology

Lung samples were cut on their largest axis and submitted to histological processing. The slides were stained with LUNA stain for the detection of eosinophils and by exclusion of neutrophils, PAS for mucus evaluation, and PSR for detection of collagen fibers. The quantitative analysis was performed using the morphometric technique described by Vieira et al. (2007) [16]. Morphological parameters were evaluated through Image Pro Plus software (version 4.5, NIH, Maryland, USA).

### 2.8. Bronchoconstriction Index

Lung samples were stored in 10% formalin for up to 7 days and then cut on their largest axis and subjected to routine histological processing. The slides were stained with HE stains to evaluate bronchoconstriction index, bronchial thickening, and airway smooth muscle thickening. Five airways of each animal were analyzed. The thickness index of the smooth muscle and bronchial epithelial layer was calculated as the number of points that focused on the smooth muscle or bronchial epithelial layer, divided by the number of intercepts that crossed the epithelial basement membrane. The bronchoconstriction index was calculated as the square root of the number of intercepts that crossed the basement membrane, divided by the number of points that focused on the lumen of the airway. The indices of the smooth muscle and epithelial layer and the bronchoconstriction index were performed in a 400x magnification [16]

### 2.9. Statistical Analysis

Data were analyzed using GraphPadPrism 5 software (USA). Data with parametric distribution were submitted to the one-way ANOVA test followed by Student Newman-Keuls test for comparison between groups. Significance levels were adjusted to 5% (p <0.05). The graphs were compiled using GraphPadPrism 5 software (USA).

## 3. Results

### 3.1. LLLT Reduces Leukocytes Evaluated in BALF

**Figure 1:**
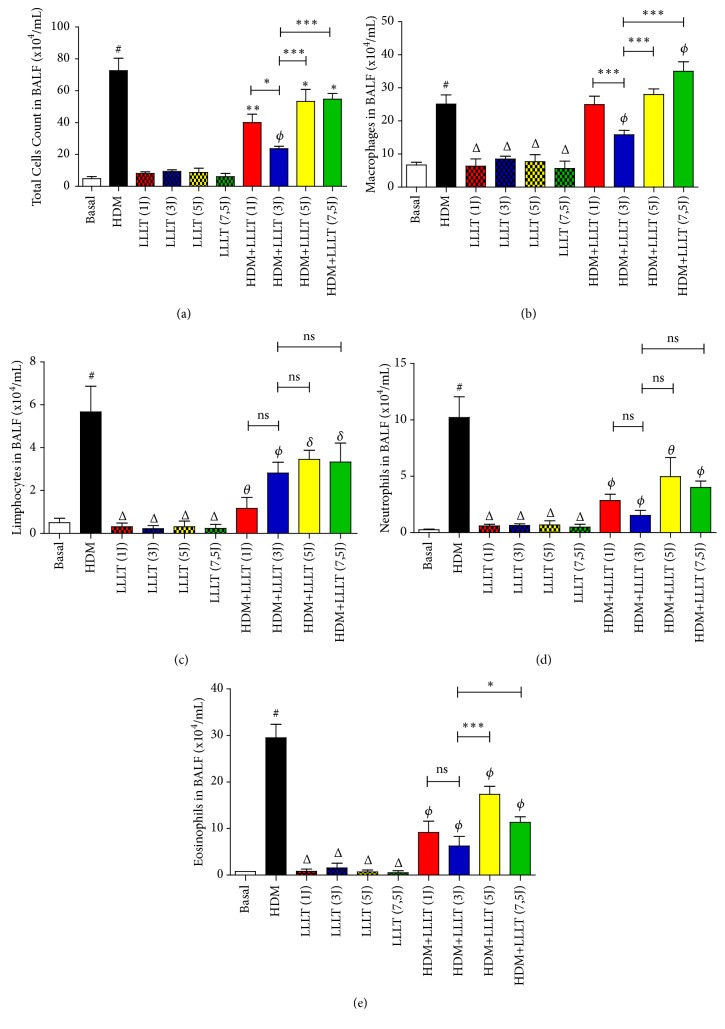
Effect of LLLT on the total number of cells (a) and the number of macrophages (b), lymphocytes (c), neutrophils (d), and eosinophils (e) recovered from the BALF. The groups used in the experiment were Basal (unmanaged animals), asthmatics (HDM) (animals immunized and challenged with HDM), LLLT (animals only treated with LLLT), and HDM+LLLT (animals sensitized and challenged with HDM and subsequently treated with LLLT). The results refer to the use of 10 mice in each experimental group. Values expressed as mean and standard deviation. # p <0.001 when compared to Basal group; *θ* p <0.001, *ϕ* p <0.01, and *δ* p <0.05 when compared to the asthmatic group (HDM) and ns (not significant); *∗* p<0,05 and *∗∗∗* p<0,001 when compared HDM+LLLT (3J) group with the other groups treated with other doses.

The results showed a significant increase in the total influx of leukocytes (Figure 1(a)), as well as in the number of macrophages, lymphocytes, neutrophils, and eosinophils (Figures 1(b), 1(c), 1(d), and 1(e)) recovered in BALF in the asthma group (HDM). On the other hand, LLLT reduced all leukocyte types studied. The results for the total number of cells and macrophage count showed a significant reduction in the HDM+LLLT (3J) group. Regarding the number of eosinophils, we observed significant results for the groups treated with the lowest doses of the laser HDM+LLLT (1J and 3J).

### 3.2. LLLT Reduces Quantification of Cytokines Proinflammatory in the BALF

**Figure 2:**
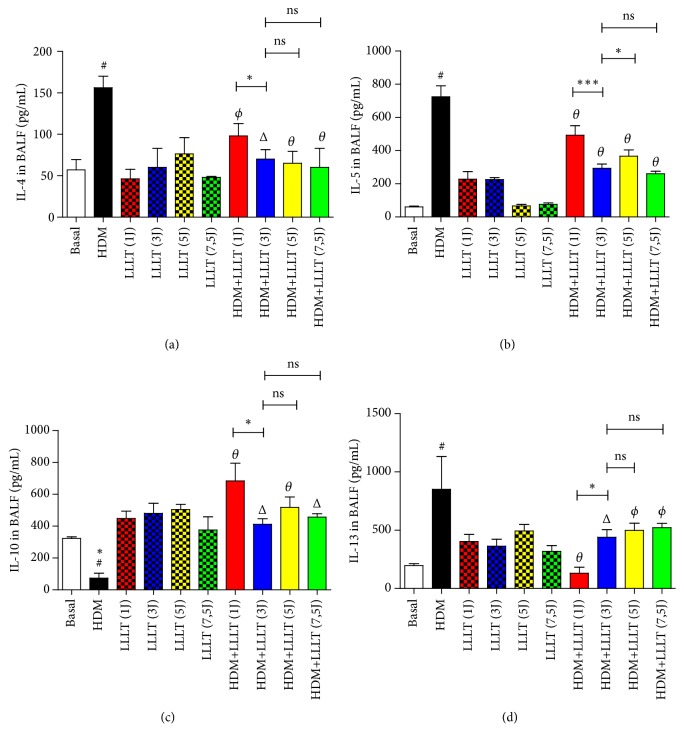
Effect of LLLT on IL-4 (a), IL-5 (b), IL-10 (c), and IL-13 (d) levels in BALF supernatant. Values expressed as mean and standard deviation. The groups used in the experiment are described in Figure 1. The results refer to the use of 10 mice in each experimental group. # p <0.001 and $\overset{*}{\#}$ p <0.01 when compared to the Basal group; *θ* p <0.001, Δ p <0.01, and *ϕ* p <0.05 when compared to the asthmatic group (HDM) and ns (not significant); *∗* p<0,05 and *∗∗∗* p<0,001 when compared HDM+LLLT (3J) group with the other groups treated with other doses.

The results showed a significant increase in the levels of the proinflammatory cytokines IL-4 (Figure 2(a)), IL-5 (Figure 2(b)), and IL-13 (Figure 2(d)) in the asthmatic group (HDM) when compared to the Basal group. We also observed a significant reduction in all asthmatic groups submitted to LLLT treatment (HDM + LLLT 1J, 3J, 5J, and 7,5J) when compared to the only asthmatic group (HDM). On the other hand, there was an increase in the anti-inflammatory cytokine IL-10 (Figure 2(c)) in all the asthmatic groups submitted to LLLT (HDM + LLLT 1J, 3J, 5J, and 7,5J) when compared to the asthmatic only group (HDM). When we evaluated the four asthmatic groups submitted to laser therapy, we observed a significant reduction of IL-13 (Figure 2(d)) and an increase in IL-10 (Figure 2(c)) for the group treated with Laser 1J (HDM + LLLT 1J).

### 3.3. LLLT Reduces Peribronchial Quantification of Eosinophils

**Figure 3:**
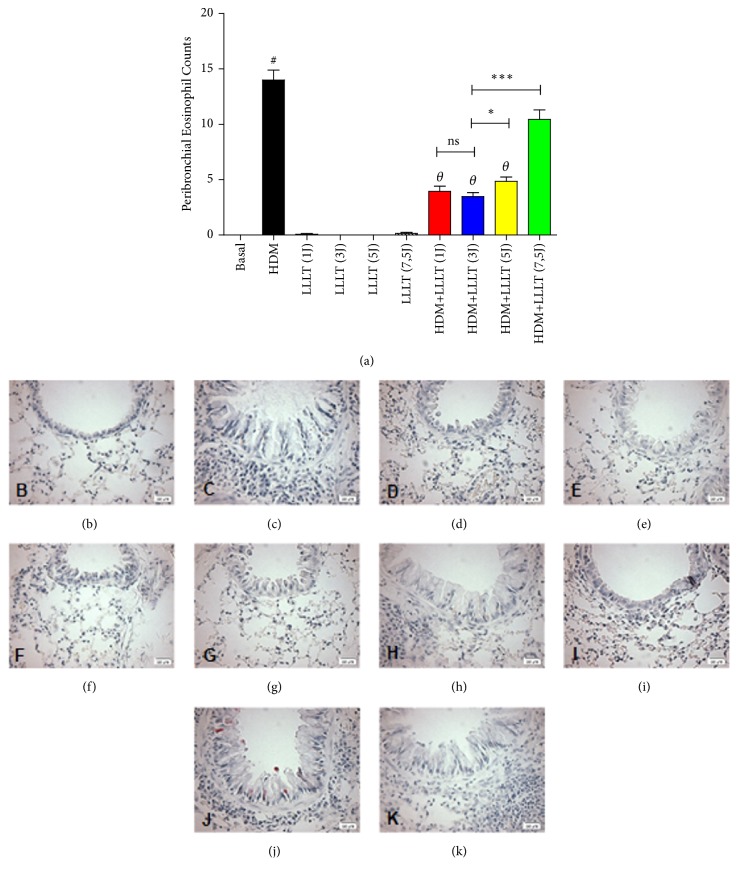
Effect of LLLT on the quantification of peribronchial eosinophils. The lungs were fixed, prepared, and stained with LUNA for the analysis of eosinophils in the airways. The animals were irradiated with LLLT 3x/week for 5 weeks, 1 hour after challenge with HDM. Increase of × 400. Values expressed as mean and standard deviation. The groups used in the experiment are described in Figure 1. The results refer to the use of 10 mice in each experimental group. # p <0.001 when compared to the Basal group and *θ* p <0.001 when compared to the asthmatic group (HDM) and ns (not significant); *∗* p<0,05 and *∗∗∗* p<0,001 when compared HDM+LLLT (3J) group with the other groups treated with other doses.

We verified a significant increase in the quantification of eosinophils in the asthmatic group (HDM) when compared to the Basal group. When comparing all the asthmatic groups that were submitted to LLLT 1J, 3J, and 5J (HDM+LLLT 1J, 3J, and 5J), we observed a significant effect on the reduction of peribronchial eosinophils quantification (Figure 3(a)). The HDM+LLLT (1J), (3J) and (5J) groups (Figure 3(a)) presented similar results among themselves, without difference of significance. The photomicrographs represent the groups used: Basal (Figure 3(b)), HDM (Figure 3(c)), LLLT (1J) (Figure 3(d)), LLLT (3J) (Figure 3(e)), LLLT (5J) (Figure 3(f)), LLLT (7,5J) (Figure 3(g)), HDM+LLLT (1J) (Figure 3(h)), HDM+LLLT (3J) (Figure 3(i)), HDM+LLLT (5J) (Figure 3(j)), and HDM+LLLT (7,5J) (Figure 3(k)).

### 3.4. LLLT Reduces Mucus in the Airways

**Figure 4:**
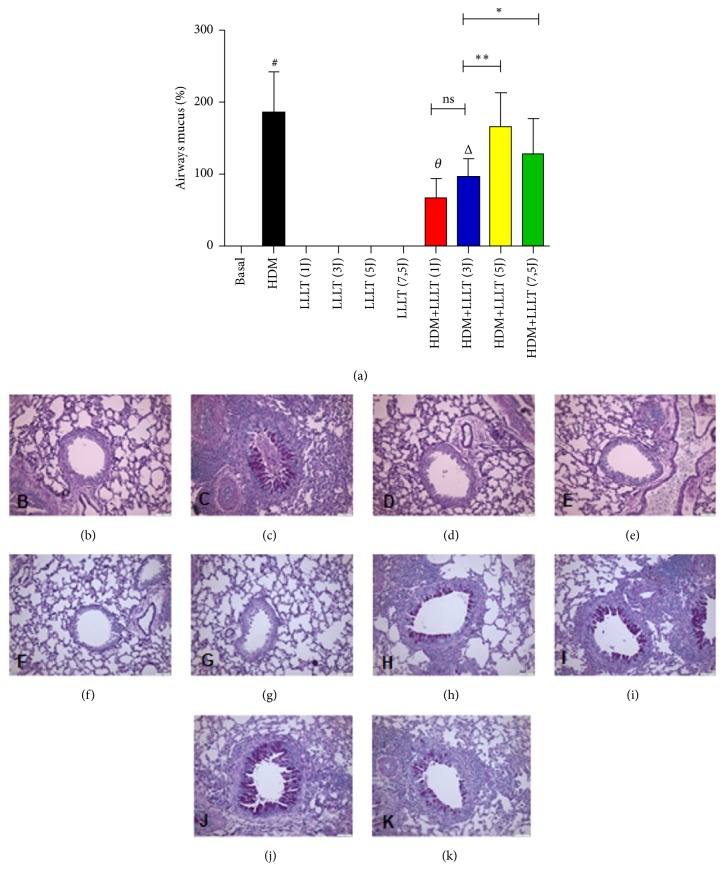
Effect of LLLT on the production of mucus in the airways. The lungs were fixed, prepared, and stained with PAS (Periodic Acid Schif) for the analysis of mucus in the airways. The animals were irradiated with LLLT 3x/week for 5 weeks, 1 hour after challenge with HDM. Increase of × 200. Values expressed as mean and standard deviation. The groups used in the experiment are described in Figure 1. The results refer to the use of 10 mice in each experimental group. # p <0.001 when compared to the Basal group and Δ p <0.01 when compared to the asthmatic group (HDM) and ns (not significant); *∗* p<0,05 and *∗∗* p<0,01 when compared HDM+LLLT (3J) group with the other groups treated with other doses.

We observed a significant increase in mucus deposition in the asthmatic group (HDM) when compared to the Basal group. When we compared all the asthmatic groups that were submitted to LLLT 1J, 3J, 5J, and 7,5J (HDM + LLLT 1J, 3J, 5J, and 7,5J) we observed a significant effect on the reduction of mucus in the airways (Figure 4(a)). We emphasize that the groups HDM+LLLT (1J) and (3J) were the two groups that presented the best results among them. The photomicrographs represent the groups used: Basal (Figure 4(b)), HDM (Figure 4(c)), LLLT (1J) (Figure 4(d)), LLLT (3J) (Figure 4(e)), LLLT (5J) (Figure 4(f)), LLLT (7,5J) (Figure 4(g)), HDM+LLLT (1J) (Figure 4(h)), HDM+LLLT (3J) (Figure 4(i)), HDM+LLLT (5J) (Figure 4(j)), and HDM+LLLT (7,5J) (Figure 4(k)).

### 3.5. LLLT Reduces Collagen in the Airways

**Figure 5:**
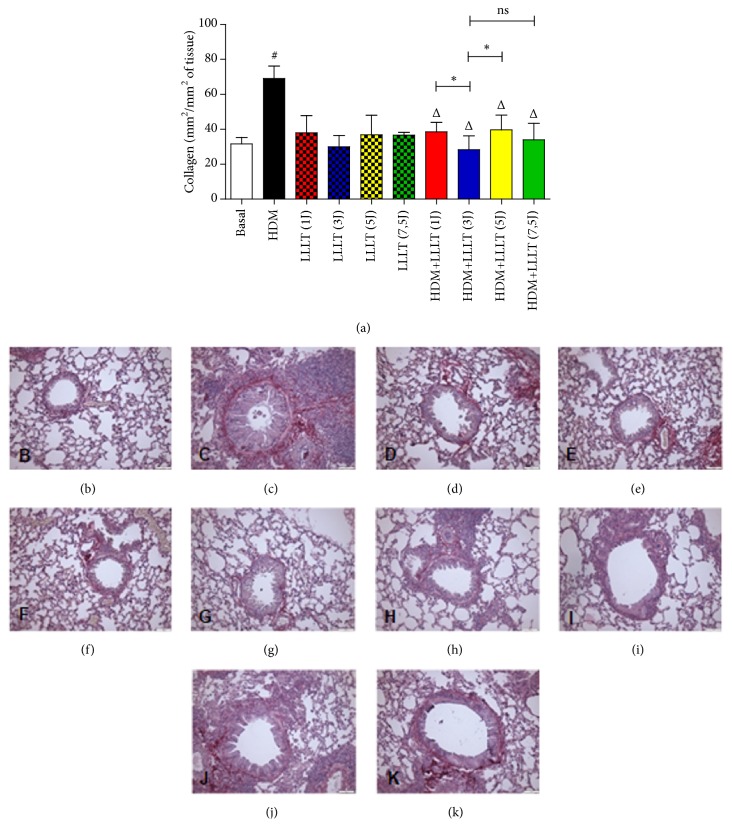
Effect of LLLT on the production of collagen in the airways. The lungs were fixed, prepared, and stained with PSR for analysis of collagen in the airways. The animals were irradiated with LLLT 3x/week for 5 weeks, 1 hour after challenge with HDM. Increase of × 200. Values expressed as mean and standard deviation. The groups used in the experiment are described in Figure 1. The results refer to the use of 10 mice in each experimental group. # p <0.001 when compared to the Basal group and Δ p <0.01 when compared to the asthmatic group (HDM) and ns (not significant) and *∗* p<0,05 when compared HDM+LLLT (3J) group with the other groups treated with other doses.

The results related to the quantification of collagen in the airways are presented below. We found a significant increase in collagen deposition in the asthmatic group (HDM) when compared to the Basal group. When we compared all the asthmatic groups that were submitted to LLLT 1J, 3J, 5J, and 7,5J (HDM + LLLT 1J, 3J, 5J, and 7,5J), we observed a significant effect on the reduction of collagen fiber deposition in the airways (Figure 5(a)). We emphasize that the groups HDM+LLLT (1J) and (7,5J) were the two groups that presented the best results among them. The photomicrographs represent the groups used: Basal (Figure 5(b)), HDM (Figure 5(c)), LLLT (1J) (Figure 5(d)), LLLT (3J) (Figure 5(e)), LLLT (5J) (Figure 5(f)), LLLT (7,5J) (Figure 5(g)), HDM+LLLT (1J) (Figure 5(h)), HDM+LLLT (3J) (Figure 5(i)), HDM+LLLT (5J) (Figure 5(j)), and HDM+LLLT (7,5J) (Figure 5(k)).

### 3.6. LLLT Reduces Bronchoconstriction Index

**Figure 6:**
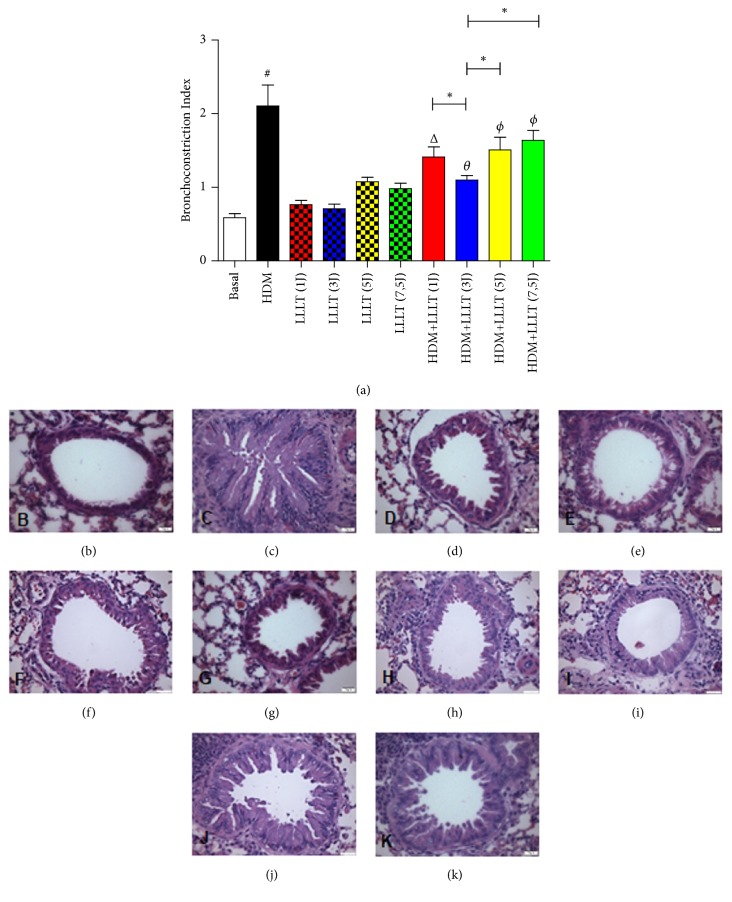
Effect of LLLT on bronchoconstriction index. The lungs were fixed, prepared and stained with HE for analysis of bronchoconstriction index in the airways. Increase of × 400. Values expressed as mean and standard deviation. The groups used in the experiment are described in Figure 1. The results refer to the use of 10 mice in each experimental group. # p <0.001 when compared to the Basal group and *θ* p <0.001, Δ p <0.01, and *ϕ* p <0.05 when compared to the asthmatic group (HDM); *∗* p<0,05 when compared HDM+LLLT (3J) group with the other groups treated with other doses.

The results related a significant increase in bronchoconstriction index in the asthmatic group (HDM) when compared to the Basal group. When we compared all the asthmatic groups that were submitted to LLLT 1J, 3J, 5J, and 7,5J (HDM + LLLT 1J, 3J, 5J, and 7,5J), we observed a significant effect on the reduction in bronchoconstriction index (Figure 6(a)). When we analyzed the groups treated among themselves, we noticed that the HDM+LLLT (3J) was significantly the one that presented the best result. The photomicrographs represent the groups used: Basal (Figure 6(b)), HDM (Figure 6(c)), LLLT (1J) (Figure 6(d)), LLLT (3J) (Figure 6(e)), LLLT (5J) (Figure 6(f)), LLLT (7,5J) (Figure 6(g)), HDM+LLLT (1J) (Figure 6(h)), HDM+LLLT (3J) (Figure 6(i)), HDM+LLLT (5J) (Figure 6(j)), and HDM+LLLT (7,5J) (Figure 6(k)).

### 3.7. LLLT Reduces Bronchial Thickening and Airway Smooth Muscle Thickening

**Figure 7:**
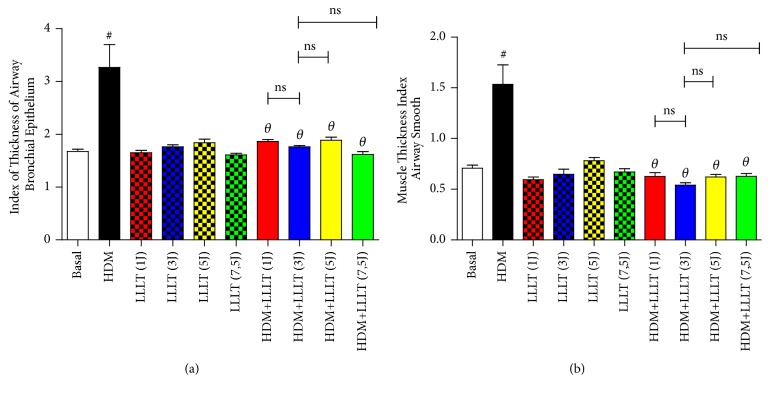
Effect of LLLT on bronchial thickening and airway smooth muscle thickening. The lungs were fixed, prepared and stained with HE for the above analyzes. Increase of × 400. Values expressed as mean and standard deviation. The groups used in the experiment are described in Figure 1. The results refer to the use of 10 mice in each experimental group. # p <0.001 when compared to the Basal group and *θ* p <0.001 when compared to the asthmatic group (HDM) and ns (not significant) when compared HDM+LLLT (3J) group with the other groups treated with other doses.

The results related a significant increase in bronchial thickening and airway smooth muscle thickening in the asthmatic group (HDM) when compared to the Basal group. When we compared all the asthmatic groups that were submitted to LLLT 1J, 3J, 5J, and 7,5J (HDM + LLLT 1J, 3J, 5J, and 7,5J), we observed a significant effect on the bronchial thickening (Figure 7(a)) and airway smooth muscle thickening (Figure 7(b)). When analyzing the groups treated among themselves, we did not notice a significant difference between them.

## 4. Discussion

The results obtained in the present study demonstrated for the first time the effectiveness of the Low-Level Laser Therapy (LLLT) in the reduction of the inflammatory cell profile, proinflammatory cytokines, peribronchial eosinophilic infiltrate, airway mucus reduction, structural improvement evaluated in the analysis bronchoconstriction index, bronchial and muscular thickening of the airway, and the quantification of collagen in an experimental model of chronic allergic lung disease induced by House Dust Mite (HDM).

Asthma is a chronic inflammatory disease that mainly affects the larger caliber structures, such as the airways. Published studies show that inflammatory cells trigger a progressive inflammatory reaction characterized mainly by eosinophil, neutrophil, and lymphocyte infiltrates, being quite present in this disease. Cells play an important role in the inflammatory or remodeling process and are directly associated with the obstructive process found in asthma [17–19].

Contact with the antigen favors cell activation and migration to the lesion site, in particular neutrophils and eosinophils. Activated T lymphocytes, on the other hand, will give rise to a lymphocyte clone with a TH2 profile and trigger the release of several proinflammatory cytokines [20]. We highlight the presence of IL-4, IL-5, and IL-13. The IL-4 cytokine has multiple and important participation in asthma, such as modulation in growth, differentiation and degranulation of mast cells, regulation of IgE synthesis by B lymphocytes, stimulation of mucus producing cells, and inhibition of TH1. On the other hand, IL-13 participates in the stimulation and maturation of B lymphocytes and the synthesis of IgE. Already IL-5 is directly involved in the recruitment of eosinophils and their longer survival time [16].

Due to the evolutionary process of the disease, there will be a variety of factors present, such as release of mediators that will trigger lesions and alterations in bronchial epithelial integrity, abnormalities in airway muscle tone, changes in mucociliary function, and increased thickening of the airway muscle as well as in its reactive response to stimuli [21]. In the later phase of the disease it is possible to observe the process of remodeling of the airways. This stage is characterized by the release of several growth factors and deposition of collagen fibers to repair the epithelial damage suffered [22].

In this way we consider it extremely relevant to study the use of LLLT in experimental models with HDM, as well as seek evidence that demonstrates its route of action and the best dose to be used, to contribute to the application of an efficient or at least auxiliary therapy, pharmacological side effects, hospital costs incurred with this disease, and morbidity and mortality.

Our results evidenced the effectiveness of photobiomodulation in reducing the inflammatory profile and structural improvement found in chronic allergic lung disease established in the current experimental model. The effects can be observed in the reduction of the cellular profile evaluated in BALF through total and differential counting, as well as in peribronchial eosinophilic infiltrate, mucus deposition, and collagen evaluated by histology. In addition, we found a decrease in IL-4 proinflammatory cytokine levels, IL-5 and IL-13 in BALF. On the other hand, the use of laser therapy increased levels of IL-10 in BALF. The bronchoconstriction of the airways showed a significant improvement in the groups treated with laser therapy, as well as for analysis of smooth muscle thickening and bronchial airway epithelium thickening.

Particularly for the evaluation of eosinophils, we observed reduction in all groups submitted to laser therapy. Data complementary to attenuation of eosinophil migration can be observed in cytokine analyzes, secifically on IL-5 levels, which are directly linked to this cell type. For the eosinophilic infiltrate, in the groups submitted to laser therapy with lower application rate, we note better results for the use of energies (1J) and (3J).

IL-4 levels were attenuated in all groups submitted to laser therapy and for the analysis of mucus quantification in the airway. The reduction of mucus deposition in the airway may contribute to a decrease in the pulmonary inflammatory process through LLLT modulation, also observed in a study published by Silva et al. (2014) and Wang et al. (2014) [9, 23].

The deposition of collagen in the airway shows a later phase, the phase of lung remodeling, in which the epithelial lesion tends to be repaired [24]. With the LLLT therapeutic intervention, especially for the HDM+LLLT (3J), we observed a positive action and reduced the deposition of the collagen fibers in the airway. In this way, LLLT was able to act positively in the prevention of structural alterations caused by chronic pulmonary inflammation.

Finally, the analyses for the bronchoconstriction index, bronchial thickening, and airway smooth muscle were significant for the groups that received LLLT treatment. Finally, the best results were obtained for the group with dose of (3J).

When we analyzed IL-10, we noticed that all groups treated with laser presented increased values, highlighting the group HDM + LLLT (1J). This suggests that IL-10, because it is an anti-inflammatory cytokine, may act to inhibit cell migration and attenuate the local inflammatory response observed in asthma.

Considering the findings of the present study, we believe that the mechanism of action of LLLT involved in inhibiting the inflammatory process of asthma may be directly linked to the activation of subpopulations of regulatory T lymphocyte (Treg), characterized by expression of FoxP3 and CD25+ [25]. Tregs cell activated secrete IL-10 that will act to control the immune response to the antigen, thereby suppressing allergic inflammation, characterized by cell migration and release of inflammatory mediators. Inhibition of the inflammatory cytokines evaluated, consequently suppress the activation and migration of eosinophils to the site of inflammation, as well as reduction of mucus production and attenuation of the return of the Th2 cells to tissues and suppress IgE production while inducing noninflammatory IgG4. Finally, Treg cells can interfere in the immune response by modulating the Th1 and Th2 response. In addition, Treg cells suppress Th2 cells and their cytokine production (IL-4, IL-5, IL-9, and IL-13), which are responsible by effector cells action in allergic lung inflammation.

To date, there is no scientific evidence that use HDM for the development of the experimental model of chronic allergic pulmonary inflammation associated with LLLT. Thus, we chose to use reference with ovalbumin (OVA), in which the animals were submitted to laser treatment [9, 23]. Taking these studies into account, we chose to develop the model with HDM, since such an agent mimics in a way that is closer to the clinical scope. The choice for such analyze considers the pathophysiological process found in asthma Finally, we consider the present study extremely relevant and important to refine the therapeutic scheme and achieve a more translational approach, helping the patients to reduce of hospitalization time and hospital expenses, as well as how to reduce pharmacological demand and side effects caused by them and promote a better quality of life for the patient.

## 5. Conclusion

We conclude in general that the use of LBI therapy in a lower dose, specifically 1J and 3J, presented significant results and better effect on the cellular profile, reduction of inflammation, and structural improvement in chronic allergic lung disease. In this way, the use of photobiomodulation acting in smaller doses for the treatment of asthma seems reasonable. We emphasize that laser is a therapeutic option that has no side effects, is low cost and noninvasive, and may have important and beneficial effects in the different phases of the disease.

## Data Availability

The all experimental data used to support the findings of this study are available from the corresponding author upon request.
